# The contribution of attentional bias to worry: Distinguishing the roles of selective engagement and disengagement

**DOI:** 10.1016/j.janxdis.2010.09.013

**Published:** 2011-03

**Authors:** Colette R. Hirsch, Colin MacLeod, Andrew Mathews, Oneet Sandher, Amruti Siyani, Sarra Hayes

**Affiliations:** a King's College London, Institute of Psychiatry, UK; b University of Western Australia, Australia; c University of California, Davis, USA

**Keywords:** Attention, Attentional engagement, Attentional disengagement, Worry, Cognitive bias modification, Anxiety

## Abstract

This study investigated the effect on worry of biased attentional engagement and disengagement. Variants of a novel attention modification paradigm were developed, designed to induce a group difference either in participants’ tendency to selectively engage with, or disengage from, threatening meanings. An index of threat bias, reflecting relative speeding to process threat word compared to non-threat word content, confirmed that both procedures were effective in inducing differential attentional bias. Importantly, when the induced group difference in attentional bias followed the procedure designed to influence selective engagement with threat meanings, it also gave rise to a corresponding group difference in worry. This was not the case when it was induced by the procedure designed to influence selective disengagement from threat meanings. These findings suggest that facilitated attentional engagement with threat meanings may causally contribute to variability in worry.

## Research highlights

▶ Two variants of a novel attention modification paradigm were developed. ▶ Attention to threat meaning was modified rather than just its location. ▶ One variant modified attentional engagement and the other disengagement from threat. ▶ Only attentional engagement training significantly influenced worry. ▶ Attentional engagement with threat meanings may causally contribute to worry.

## 1 Introduction

Worry is a central cognitive symptom of anxiety observed across many anxiety disorders, and uncontrollable worry is the key defining feature of generalized anxiety disorder (GAD; American Psychiatric Association, 1994). Heightened anxiety vulnerability is characterized by a selective attentional bias favouring threatening information, which some clinical theorists contend makes a causal contribution to anxiety symptomology, such as excessive worry (cf. Mathews & MacLeod, 1994; Mathews & MacLeod, 2005). This attentional bias has been demonstrated using a range of different tasks, including the emotional Stroop task (cf., Williams, Watts, MacLeod, & Mathews, 1997), and the dot-probe task (e.g., MacLeod & Mathews, 1988; MacLeod, Mathews & Tata, 1986; Mogg, Mathews, & Eysenck, 1992).

There has been recent debate concerning whether elevated anxiety vulnerability reflects facilitated attentional engagement with, or impaired subsequent disengagement from, threatening information. Some evidence suggests that such vulnerability is associated with speeded attentional engagement with the locus of threatening information (Garner, Mogg & Bradley, 2006; Koster, Crombes, Verschuere, Damme, & Wiersema, 2006). However, there also is evidence that it is associated with a slowing to disengage attention from the locus of such information (Amir, Elias, Klumpp & Przeworski, 2003; Fox, Russo, Bowles & Dutton, 2001; Koster, Crombez, Verschuere, & De Houwer, 2004; Salemink, van den Hout & Kindt, 2007; Yiend & Mathews, 2001). On occasions, researchers have found evidence of both attentional effects in anxious participants (Mathews, Fox, Yiend & Calder, 2003). While this suggests that facilitated engagement with threat, and impaired disengagement from threat, may both be associated with anxiety vulnerability, such findings do not reveal whether the engagement bias, the disengagement bias, or both, contribute causally to anxiety symptoms such as worry.

Though not resolving this specific issue, the recent use of cognitive bias modification procedures has established that biased attention does contribute to worry. Recent studies using moderate and high level worriers (Hayes, Hirsch, & Mathews, 2010; Krebs, Hirsch, & Mathews, 2010) employed an attention modification approach, based on that introduced by MacLeod, Rutherford, Campbell, Ebsworthy and Holker (2002), to demonstrate that attentional bias causally impacts on worry. For example, Hayes et al. (2010) randomly assigned high worriers to either a training condition in which they repeatedly directed their attention away from the source of worry-related information and towards benign information, or to a control condition not designed to modify attention. This procedure served to induce a group difference in attentional bias, such that the former participants came to display significantly less evidence of selective attention to threat than did the latter. All participants then reported on thought intrusions while focusing on their breathing, before and after an instructed worry period (Borkovec, Robinson, Pruzinsky, & De Pree, 1983; Ruscio & Borkovec, 2004). Again a significant group difference was evident, with those individuals exposed to benign attentional training reporting less negative thought intrusions than control participants.

While these findings suggest that attentional bias makes a causal contribution to worry, they do not reveal whether it is enhanced attentional engagement with threat, impaired attentional disengagement from threat, or both, that exerts this causal impact. To address this issue, it would be necessary to examine whether inducing a group difference in attentional engagement with threat, or inducing a group difference in attentional disengagement from threat, each serve to produce a corresponding group difference in negative thought intrusions, during a subsequent worry assessment task.

It is important to note that attention to the spatial locus of a threat stimulus is not necessarily the same as attention to the threatening meaning of this stimulus, and it is selective attention to threat meanings that is assumed to be the critical causal factor in anxiety. Moving attention toward a given spatial locus does not ensure the semantic processing of stimuli in that region, just as moving attention away from a previously attended spatial locus does not preclude the continued semantic processing of information presented there. For example, non-anxious individuals may avoid focusing on the threatening meaning of a spatially attended stimulus, while anxious individuals may not only process the threat meaning but also continue to focus on it even after shifting attention away from the stimulus location. In this way, an external threat cue could trigger worry, involving attention to internally maintained threatening meanings, which then continues long after disengagement from the location of the original cue.

To determine the contribution to worry made by biased attention to threat meanings, a methodology is required that can influence the ease with which attention can be shifted selectively towards or away from processing threatening meanings. In the present study, this was achieved using a novel procedure that required participants to switch between two different types of decision concerning the same stimulus word; an affective decision requiring semantic access (good or bad?), and a non-semantic structural decision (upper or lower case letters?).

By employing two alternative variants of this procedure, we sought to induce a group difference in selective attention to threat in either one of two quite different ways. One approach was designed to induce such a difference through training biased selective attentional engagement with threat meanings. This involved participants repeatedly switching from structural to affective decisions for one particular valence of stimuli, with some participants being required to consistently make this switch only for threat words (to encourage selective engagement with threat meanings), and others being required to consistently make this switch only for non-threat words (to discourage selective engagement with threat meanings). The other approach was designed to induce a group difference in selective attention to threat through training biased attentional disengagement from threat meanings. This involved participants repeatedly switching from affective to structural decisions for one particular valence of stimuli, with some participants being required to consistently make this switch only for threat words (to encourage selective disengagement from threat meanings), and others being required to consistently make the switch only for non-threat words (to discourage selective disengagement from threat meanings).

If group differences in attention to threat can be successfully induced in each of these two ways then, by comparing such groups in terms of subsequently reported negative thought intrusions, it will be possible to determine whether differential attentional engagement with threat, and/or differential attentional disengagement from threat, causally contribute to differential worry.

## 2 Method

### 2.1 Design

The study involved two consecutive phases: an attentional bias induction phase and a worry assessment phase. Participants were assigned at random either to a training procedure designed to induce a group difference in engagement bias, or to a training procedure designed to induce a group difference in disengagement bias. In each case, half the participants were exposed to a condition intended either to encourage, or to discourage, the selective processing of threatening meanings. Thus, participants were randomly allocated to one of the four attentional bias induction conditions: (1) increase threat processing by encouraging selective engagement with threat meanings; (2) increase threat processing by discouraging selective disengagement from threat meanings; (3) decrease threat processing by discouraging selective engagement with threat meanings; or (4) decrease threat processing by encouraging selective disengagement from threat meanings. In all cases, participants were required to make two consecutive decisions about each target word, either concerning its structure (i.e. upper or lower case of the letters) or its semantic content (affective valence), but these decisions varied according to attentional bias induction condition (see Table 1).

Following the attention bias induction phase, all participants completed Hayes et al.’s (2010) worry task, which involved categorising the valence of thought intrusions that occurred before and after an instructed worry period. Participants subsequently provided expanded descriptions of these thought intrusions, enabling an assessor, who was not informed of group allocation, to categorise their valence.

### 2.2 Participants

Sixty-four student participants were selected based on their scores on the Penn State Worry Questionnaire (PSWQ; Meyer, Miller, Metzger, & Borkovec, 1990). Due to the concern that inducing selective attention to threat meanings might have adverse effects on very high trait worriers, only participants who scored 55 or below on the PSWQ were invited to take part in the study (Molina & Borkovec, 1994). Of the 64 volunteers, 16 were randomly allocated to each of the following training conditions: (i) encourage selective engagement with threat meanings (3 males and 13 females); (ii) discourage selective disengagement from threat meanings (9 males and 7 females); (iii) discourage selective engagement with threat meanings (7 males and 9 females); and (iv) encourage selective disengagement from threat meanings (4 males and 12 females). Note that conditions (i) and (ii) both are designed to induce attentional bias that favours the processing of threat meanings, while conditions (iii) and (iv) instead are designed to induce attentional bias that compromises processing of threat meanings. The ratio of males to females selected for each group did not significantly differ, *χ*^2^(3, *N* = 64) = 6.18, *ns*. Groups also did not differ significantly in scores on the PSWQ (*M* = 41.7; *SD* = 7.3; *F*(3, 60) = 2.20, *ns*.) or State-Trait Anxiety Inventory Trait version (STAI-T; Spielberger, Gorsuch, Lushene, Vagg, & Jacobs, 1983; *M* = 39.3; *SD* = 5.9; *F* < 1). Average age was 19.5 (*SD* = 2.7), with no significant age difference between groups, *F* < 1.

### 2.3 Materials

#### 2.3.1 Experimental stimuli

A set of 96 words (48 threat and 48 non-threat) was selected from an initial pool of 377 words, based on ratings provided by ten independent judges. These judges rated each item on a 7-point scale (1 = very positive, 7 = very negative). The threat words selected for use all had a mean rating above 6.00 (*M* = 6.44, *SD* = 0.70), while the non-threat words all had a mean rating below 3.00 (*M* = 1.76, *SD* = 0.77). Of the final set of 96 words, 64 were assigned to attention modification trials and 32 to test trials.

#### 2.3.2 Emotional assessment instruments

Trait worry level was measured using the Penn State Worry Questionnaire (PSWQ; Meyer et al., 1990), a 16-item measure consisting of statements about worry (e.g., “Once I start worrying, I can’t stop”), each with a 5-point answer scale ranging from 1 (not at all typical of me) to 5 (very typical of me) yielding a total score ranging from 16 to 80, with higher scores indicating greater worry levels. The PSWQ has good psychometric properties in student, community, and clinical samples, with studies reporting high internal consistency, short-term retest reliability, and convergent and criterion related validity (Brown, Antony, & Barlow, 1992; Davey, 1993).

Trait anxiety was measured using the State-Trait Anxiety Inventory (STAI-T; Spielberger et al., 1983), consisting of 20 anxiety symptoms that participants rate for frequency of occurrence. Scores range between 20 and 80, with a higher score indicating greater anxiety. The STAI-T has good internal consistency (.89) and test–retest reliability (.88; Barnes, Harp, & Jung, 2002).

#### 2.3.3 Attentional bias induction tasks

These tasks required participants to switch attention between the structure and the semantic content of words, by making two sequential decisions about every word relating either to the form of the letters in which it was written (i.e. whether the word was presented in “big letters”/“upper case” or “small letters”/“lower case”), or to its emotional meaning (i.e. whether the word was “positive”/“good” or “negative”/“bad” in meaning).^1^

On each trial a fixation cross appeared in the centre of the screen for 1500 ms, after which the first question appeared (e.g., ‘Big?’). After 1000 ms this was replaced by a target word (e.g., CANCER) that appeared for 400 ms. Participants were instructed to quickly answer the first question correctly, by pressing the corresponding “y” (yes) or “n” (no) key. Incorrect responses were signaled by a short error tone. Immediately following registration of the participant's response, the second question about the word appeared (e.g., ‘Positive?’). The next trial commenced 1500 ms after the participant responded to the second question.

The exact sequence of events within trials varied according to participant group (see Table 1 for a summary of the different bias induction conditions). Half the participants were exposed to a procedure designed to induce a group difference in selective attentional engagement with threat meanings. For these participants the first decision always concerned the structure of the word, to ensure that initially attention was focused on this non-semantic aspect of the stimulus. The participant then was immediately required to make a second judgment about the stimulus, which could concern either structure again, or semantic content. For the subset of such participants exposed to the condition designed to encourage selective engagement with threat meanings, this second decision always concerned valence when the target word was threatening, and structure when it was non-threatening. Thus, making this second decision required these participants to move attention from stimulus structure to selectively engage with the meanings of threatening, but not non-threatening words. For the other subset of such participants, who were exposed to the condition designed to discourage selective engagement with threat meanings, the second decision always concerned valence when the target word was non-threatening, and structure when it was threatening. Thus, making this second decision required these participants to move attention from stimulus structure to selectively engage with the meanings of non-threatening, but not threatening words.

The remaining participants were assigned to a procedure designed to induce a group difference in selective attentional disengagement from threat meanings. These participants always were required to make their first decision about the valence of each word, to ensure that attention was initially focused on stimulus meaning. For the subset exposed to the condition designed to discourage selective disengagement from threat meanings, the second decision always concerned stimulus valence when the word was threatening, and structure when the word was non-threatening. Thus, making this second decision required selective disengagement from the content of non-threatening words, but not from the content of threat words. Finally, for those participants exposed to the condition designed to encourage selective disengagement from threat meanings, the second decision concerned structure when the word was threatening, and valence when the word was non-threatening. Thus, making this second decision required these participants to selectively disengage attention from the content of threat words, but not from the content of non-threatening words.

The task delivered a total of 256 attention bias induction trials, across four consecutive blocks of 64 such trials, with each word presented once per block in random order. Participants were offered a rest break after each block. Participants also completed 32 attentional bias assessment trials, 16 using new threat words and 16 using new non-threat words. In these assessment trials, although the first decision about each stimulus was of the type required in the bias induction trials completed by the participant, the second decision always concerned stimulus valence. The degree to which participants were relatively faster to make this content decision on threat words, compared to non-threat words, on these assessment trials, provided an index of attentional preference for threat meanings. This made it possible to determine whether the bias induction procedures did induce a group differences in attentional bias.

### 2.4 Filler task

The Speed of Comprehension Test (Version A; Baddeley, Emslie, & Nimmo-Smith, 1992) was administered as a filler to reduce the likelihood of differences in mood prior to the worry task. Participants decided whether 100 sentences were true or false for 2 min. To minimize anxiety instructions emphasized that speed was not important.

### 2.5 Worry task

This task was based on that adopted by Hayes et al. (2010), itself based on the method developed by Borkovec et al. (1983). There were three phases: a 5 min pre-worry induction breathing focus period, which assessed tendency to worry prior to its active experimental induction; a 5 min period of worrying; and a further 5 min post-worry induction breathing focus period, which assessed the persistence of the induced worry. During each breathing focus period participants were instructed to focus their attention on their breathing. Twelve tones were presented at random intervals of 20–30 s (Donaldson, 2004), signaling participants to report if their attention was focused on their breathing, or if they were experiencing a thought intrusion. If the latter, they indicated whether it was positive, benign, or negative, and provided a brief description (e.g., “positive – going on holiday”).

After the pre-worry breathing focus period, participants identified a current worry topic which was discussed briefly with the experimenter to ensure that it was related to a potentially negative future situation. They were then asked to continue to silently worry about this topic for 5 min, and the experimenter left the room. After 5 min the experimenter returned and the post-worry breathing focus period was completed.

Finally, participants were asked to expand on the thought intrusions reported during the breathing focus periods. For each intrusion, the experimenter read aloud the participant's summary, and asked them to describe what was going through their minds at the moment they originally had that thought. Descriptions were recorded for later rating by a psychologist, who assessed the valence of each intrusion. To assess reliability, a psychology student also rated 16 participants’ thought expansions. Neither assessor was informed about group allocation, nor when the intrusion occurred. Inter-rater reliability for assessors’ valence ratings using Cohen's Kappa statistic (*k*) was 0.89.

### 2.6 Procedure

Participants initially completed the PSWQ and STAI-T. After instructions and 16 practice trials, the attentional bias induction task was administered. This was followed by the filler task. Participants then received instructions for the worry task, practiced focusing on their breathing and providing a capsule summary of their thoughts at three random time intervals within 45 s. When it was clear that all instructions were understood, participants were administered the worry task. Finally participants were debriefed and paid £15 ($22 USD) or given course credit.

## 3 Results

### 3.1 Data analytic plan

Data from the attentional bias induction task was first analyzed for accuracy to determine if there were any group differences. Then the reaction time data from the assessment trials were investigated in the following way. To determine whether the task was successful in inducing differential attentional bias to threat as intended, the median response latencies for the second (valence) decision were calculated for each participant. Then a threat bias index was computed for each participant, reflecting relative speeding to make this content decision on threat words compared to non-threat words (by subtracting median latency on threat words from median latency on non-threat words). A higher score on this threat bias index reflects the facilitated processing of threat word content relative to non-threat word content, consistent with selective attentional bias to threatening information. The threat bias index was analyzed by examining the effects of Modification Type (engagement vs. disengagement) and Modification Direction (increase threat processing vs. decrease threat processing) to determine whether the targeted attentional biases had been induced. Finally the negative thought intrusion data was analyzed to determine the effects of Modification Type and Modification Direction, Time (pre- vs. post-worry induction) and Rater (participant vs. assessor).

### 3.2 Attentional bias induction task

#### 3.2.1 Accuracy

Accurate responses to target words averaged 95.8% (*SD* = 2.8) on training trials, and 96.1% (*SD* = 2.7) on assessment trials, with no significant difference in accuracy between the four groups (*F* < 1; *F*(3,63) = 1.03, *ns*). Hence, any group differences on assessment trials could not be attributed to differential speed-accuracy trade-offs.

#### 3.2.2 Assessment trials

A two-way ANOVA was carried out on these threat bias index scores that considered the between-group factors: Modification Type (Engagement vs. Disengagement); and Modification Direction (increase threat processing vs. decrease threat processing). See Table 2 for means and standard deviations. As expected, this analysis revealed a significant main effect of Modification Direction, *F*(1,60) = 15.52, *p* < .001, *f*^2^ = 0.50. This reflected the fact that participants in the decrease threat processing groups obtained lower threat bias index scores than did those in the increase threat processing groups (*M* = −130.64, *SD* = 166.54, vs. *M* = 42.92, *SD* = 184.71). Therefore, as intended, the bias induction procedure did indeed appear to induce group differences in selective attention to threat meanings. Importantly, Modification Type did not influence attentional bias index scores, either as a main effect *F*(1,60) = 1.34, *ns*., *f*^2^ = 0.15, or in an interaction involving Modification Direction, *F* < 1. Hence the procedures designed to induce a group difference in attentional bias through the manipulation of selective engagement with, and selective disengagement from, threat meanings were equally effective in inducing such a group difference in attentional bias. Indeed, *t*-tests on threat bias index scores, revealed similar effects of Modification Direction, in terms of the resulting magnitude of group difference in attentional bias, whether the induction procedure targeted engagement bias (*t*(30) = 2.96, *p* < .01, *d* = 1.08) or disengagement bias (*t*(30) = 2.60, *p* < .05, *d* = 0.95).

### 3.3 Worry task

The number of negative thought intrusions recorded during each breathing focus period, as categorized by participants and assessor, are presented in Table 3. These data were analyzed in a mixed-model ANOVA with two between-participants factors, Modification Type and Modification Direction, and two repeated measures factors, Time (pre- vs. post-worry induction) and Rater (participant vs. assessor). This analysis revealed a main effect of Time, *F*(1,60) = 9.89, *p* < .005, *f*^2^ = 0.40, with more negative thought intrusions post- than pre-worry induction (*M* = 1.95 vs. *M* = 1.35). There was also a main effect of Rater, *F*(1,30) = 4.76, *p* < .05, *f*^2^ = 0.28, with participants themselves identifying more negative thought intrusions than the assessor (*M* = 1.76 vs. *M* = 1.55). More critically, a significant interaction now was obtained between Modification Type and Modification Direction, *F*(1,60) = 5.15, *p* < .05, *f*^2^ = 0.29, which was the only other significant effect to emerge from the analysis. This interaction was not further qualified by Time (*F*(1,30) = 1.12, *ns*.), suggesting that the impact of modifying attentional selectivity was not restricted to the persistence of worry only after it was experimentally induced, but was equally evident on the measure of tendency to worry taken prior to the experimental worry induction. Nor was it modified by Rater (*F* < 1), indicating that it did not reflect the impact of bias induction on participants’ criteria for classifying the valence of thought intrusions.

The interaction between Modification Type and Modification Direction was investigated further. For participants exposed to the procedure designed to create a group difference in attentional bias through inducing discrepant patterns of selective disengagement from threat, there was no effect of Modification Direction on negative thought intrusions, with a similar number of such negative intrusions being evidenced across the conditions designed to decrease or to increase threat processing (*M* = 1.92, *SD* = 1.38, vs. *M* = 1.67, *SD* = 1.76 *t* < 1). In contrast, for participants exposed to the procedure designed to create differential attentional bias through inducing discrepant patterns of selective engagement with threat, there was a strong effect of Modification Direction on negative thought intrusions. Specifically, the condition designed to decrease threat processing (discourage engagement with threat meanings) now led to fewer negative intrusions than did the condition designed to increase threat processing (encourage engagement with threat meanings; *M* = 0.83, *SD* = 0.62 vs. *M* = 2.19, *SD* = 1.63; *t*(30) = 3.12, *p* < .01, *d* = 1.14). Thus, when the bias induction procedure served to induce a group difference in selective attention to threat through a contingency designed to affect attentional engagement with threat, then it also served to create a corresponding group difference in worry symptomatology, which was not the case when differential attention to threat instead was induced through a contingency designed to affect attentional disengagement from threat.

## 4 Discussion

This study introduces a novel attentional bias modification method, designed to induce group differences in selective attention to threat meaning, rather than only to the spatial location of threat stimuli. Participants who completed attentional induction procedures designed to decrease processing of threat meanings were slower to process the semantic content of threat words relative to non-threat words than were participants assigned to induction procedures designed to increase threat processing. This effect was equally evident whether the bias induction procedure involved training that targeted biased attentional engagement with, or biased attentional disengagement from, threat meanings. However, while these different induction bias procedures were equally effective in inducing differential selective attention to threat meanings, they had quite different effects on subsequent patterns of worry.

When the attentional bias induction procedure encouraged selective attentional engagement with threat meanings, the frequency of negative thought intrusions on the worry task was greater than when the procedure was designed to discourage selective engagement with threat meanings. In contrast, no such differences in negative thought intrusions were found between the attentional bias induction procedures designed to encourage or discourage selective attentional disengagement from threat meanings. These findings are not readily attributable to differences in performance accuracy between the alternative attention induction tasks, since groups did not differ in these respects. Rather, this pattern of results indicates that variability in selective attentional engagement with threat, but not variability in attentional disengagement from threat, causally impacts on negative thought intrusions indicative of worry. Consequently we suggest that biased attentional engagement with threat meanings influences the tendency to worry, perhaps by affecting the degree to which selective attention is drawn towards those negative aspects of the situation that serve to trigger negative thoughts.

Previous research has yielded evidence that elevated anxiety vulnerability is associated with both biased attentional engagement with, and biased attentional disengagement from, the spatial locus of threatening stimuli. As noted earlier, such patterns of attention to spatial loci need not imply that anxiety vulnerability is characterized by both biased attentional engagement with, and disengagement from, threat meanings. Furthermore, even if anxiety vulnerability is found to be associated with both increased engagement with and impaired disengagement from threat meanings, this would not permit the inference that each form of attentional bias contributes to anxious symptomatology. Addressing this issue requires the development of procedures designed to induce either differential selective engagement with or disengagement from threatening meanings, in order to reveal their respective effects on anxiety symptoms. The present research has adopted this approach and, at least with respect to worry, suggests that biased attentional engagement with threatening meanings may be more critical than biased attentional disengagement from such information in causally contributing to worry-related negative thought intrusions. Future research should examine whether this remains true when people are already engaged in worrying and in clients with Generalized Anxiety Disorder.

In conclusion, we report the first attempt to induce differences in selective attentional engagement with, and in selective attentional disengagement from, threat meanings, in order to distinguish the causal contributions made by each pattern of attentional selectivity to worry. A procedure designed to induce differential selective attentional engagement with threat meanings served to influence negative thought intrusions, whereas a procedure designed to induce differential attentional disengagement from threat meanings did not, despite the fact that both procedures succeeded equally in inducing differential attentional bias. It would appear that enhanced attentional engagement with threat meanings may causally contribute to negative thought intrusions indicative of worry.

## Figures and Tables

**Table 1:** Outline of the design showing the first and second decision task for threat and non-threat words in each condition.

Bias induction direction	Condition	Decision 1	Decision 2
Increase threat processing	Encourage threat engagement	Word structure	Valence for threat or structure for non-threat
	Discourage threat disengagement	Word valence	Valence for threat or structure for non-threat

Decrease threat processing	Discourage threat engagement	Word Structure	Structure for threat or valence for non-threat
	Encourage threat disengagement	Word valence	Structure for threat or valence for non-threat

*Note*: The second decision (about its valence or structure) varied by word type (threat or non-threat). For example, the encourage threat engagement condition involved an initial structural decision, followed by an emotional valence decision (good or bad?) for threat words but a structural (e.g. upper or lower case letters?) decision for non-threat words, thus requiring a shift to engagement with emotional meaning for threat but not non-threat words.

**Table 2:** Median attention modification test trial response latencies and threat bias index for each condition (standard deviations in parentheses).

Modification Direction	Condition	Trial valence		Threat bias index
		Non-threat	Threat	
Increase threat processing	Encourage threat engagement	1017.50 (225.47)	986.2500 (221.52)	31.25 (180.65)
	Discourage threat disengagement	1007.53 (237.41)	952.94 (126.42)	54.59 (193.87)

Decrease threat processing	Discourage threat engagement	913.31 (142.89)	1083.34 (245.57)	−170.03 (202.86)
	Encourage threat disengagement	916.25 (166.80)	1007.50 (162.87)	−91.25 (113.38)

**Table 3:** Mean number of negative thought intrusions for each condition during the worry task pre-worry and post-worry periods, as rated by self and assessor (standard deviations in parentheses).

Modification Direction	Condition	Pre-worry		Post-worry	
		Self	Assessor	Self	Assessor
Increase threat processing	Encourage threat engagement	2.00 (1.37)	1.69 (1.19)	2.94 (2.89)	2.12 (2.42)
	Discourage threat disengagement	1.50 (1.41)	1.12 (1.50)	2.06 (2.23)	2.00 (2.22)

Decrease threat processing	Discourage threat engagement	0.94 (1.12)	0.69 (0.70)	0.87 (0.88)	0.81 (0.98)
	Encourage threat disengagement	1.44 (1.31)	1.44 (1.36)	2.31 (1.74)	2.50 (1.93)

## Footnotes

1 Two sets of labels were used for each question type, such that two sequential questions concerning structure (or valence) always differed even if they related to the same concept (e.g., “big letters?” followed by “upper case?”, or “positive?” followed by “good?”).
